# Supplementary material for: The Landscape of Accessible Chromatin and Developmental Transcriptome Maps Reveal a Genetic Mechanism of Skeletal Muscle Development in Pigs
Source: Int J Mol Sci. 2023 Mar 29;24(7):6413. doi: 10.3390/ijms24076413 (PMC10094211; doi:10.3390/ijms24076413)
Supplement: Supplementary file 1 [file ijms-24-06413-s001.zip › ijms-2277085-supplementary.pdf]

S1

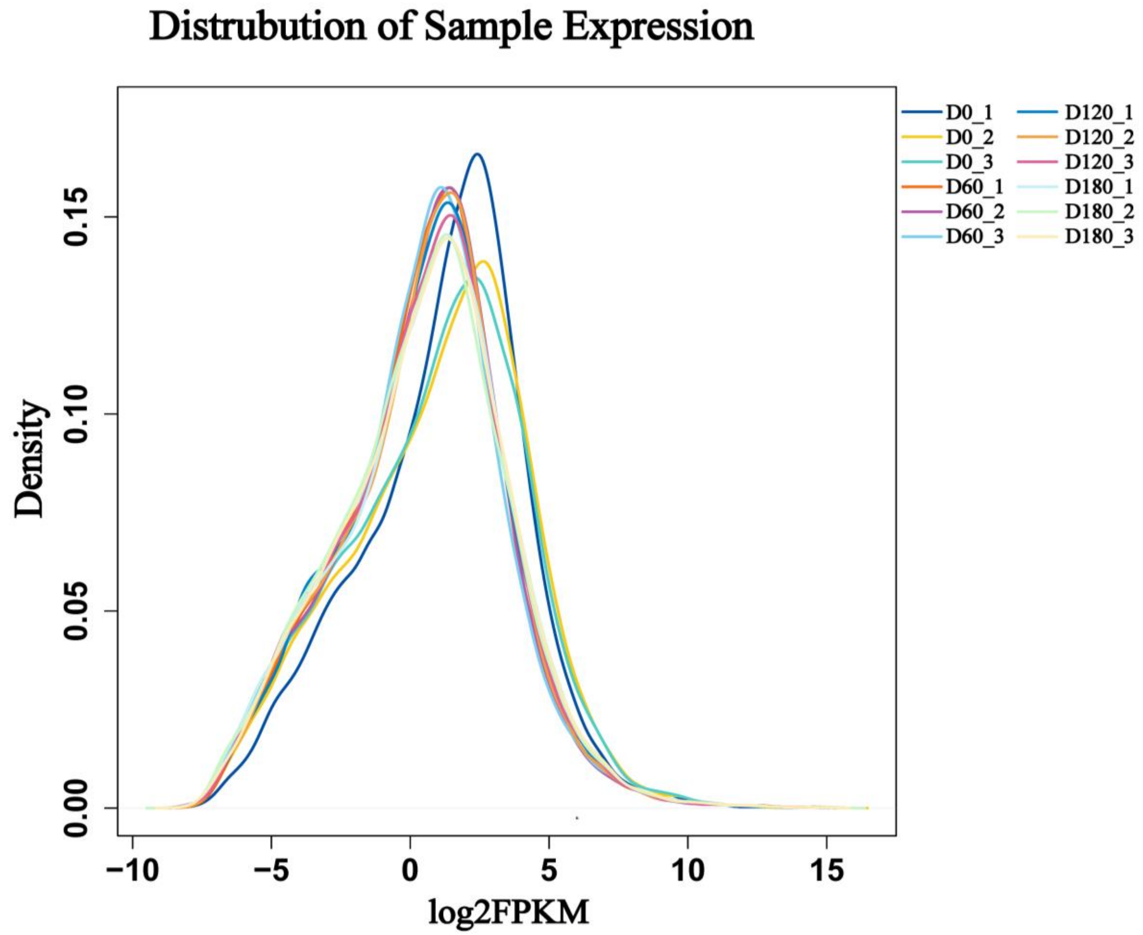

Figure S1. Density distribution map of gene expression in each sample. (Nore:The abscissa is log2(FPKM), which represents the logarithm of gene expression in each sample. The higher the value, the higher the gene expression. The ordinate is the density of genes, different colors represent different samples, and the peak of the density distribution curve represents the area where the gene expression concentration of the whole sample is concentrated.).

S2

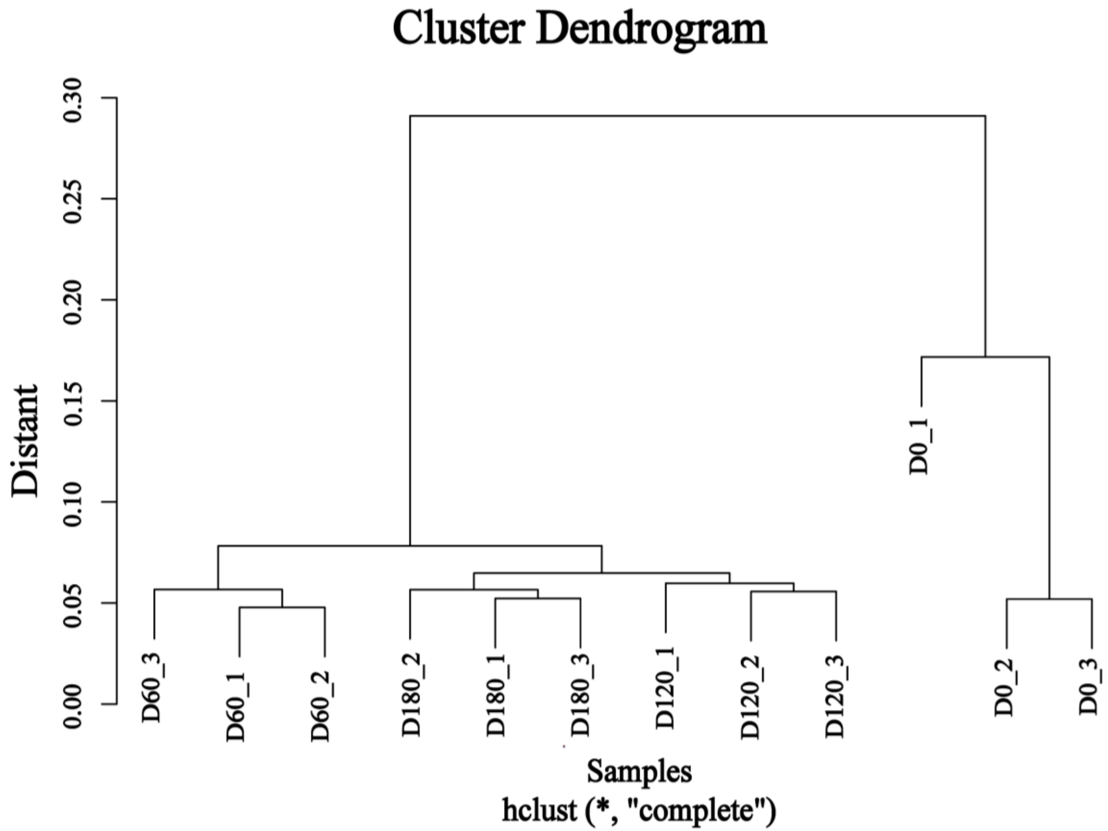

Figure S2. Clustered dendrogram of 12 samples of skeletal muscle from Landrace pigs.

S3

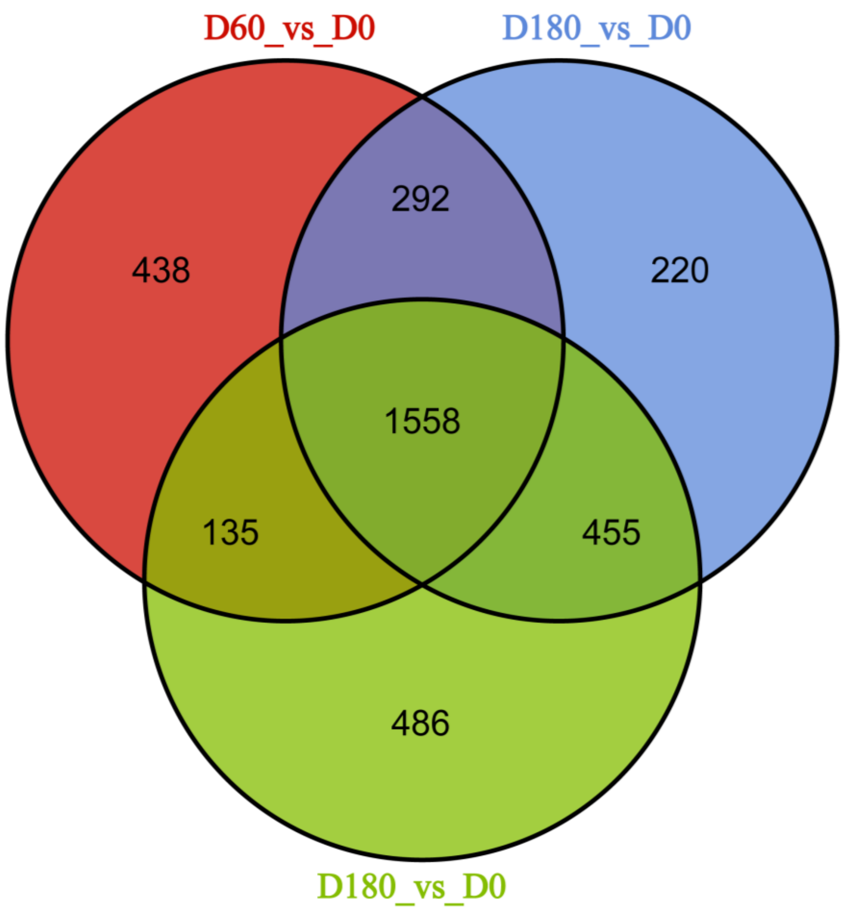

Figure S3 Venn diagram of three groups D60vsD0, D120vsD0, and D180vsS0 of landrace pig skeletal muscle.

S4

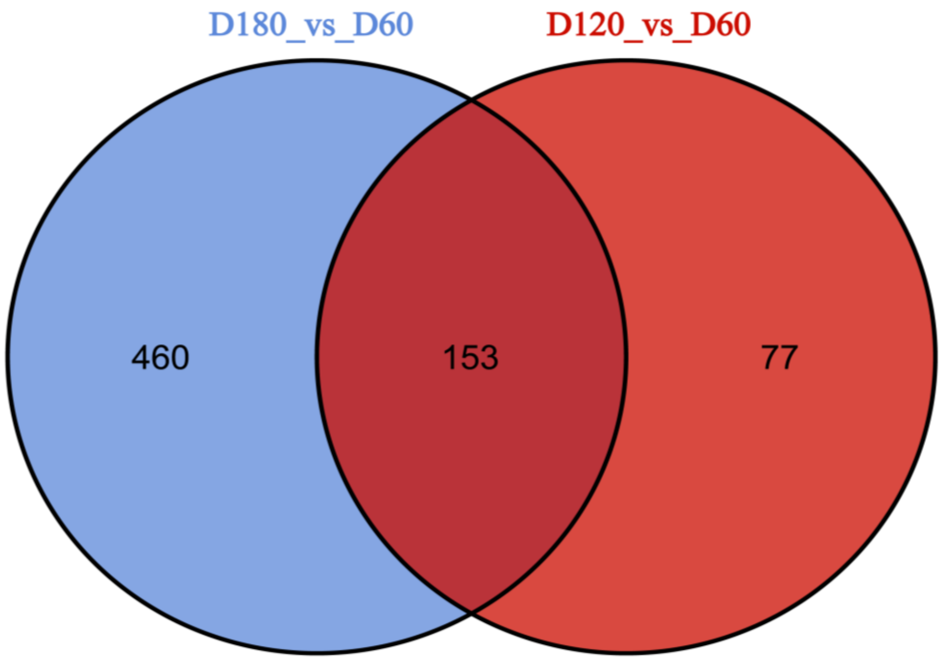

Figure S4. Venn diagram of two groups D180vsD60, D120vsD60, of landrace pig skeletal muscle.

S5

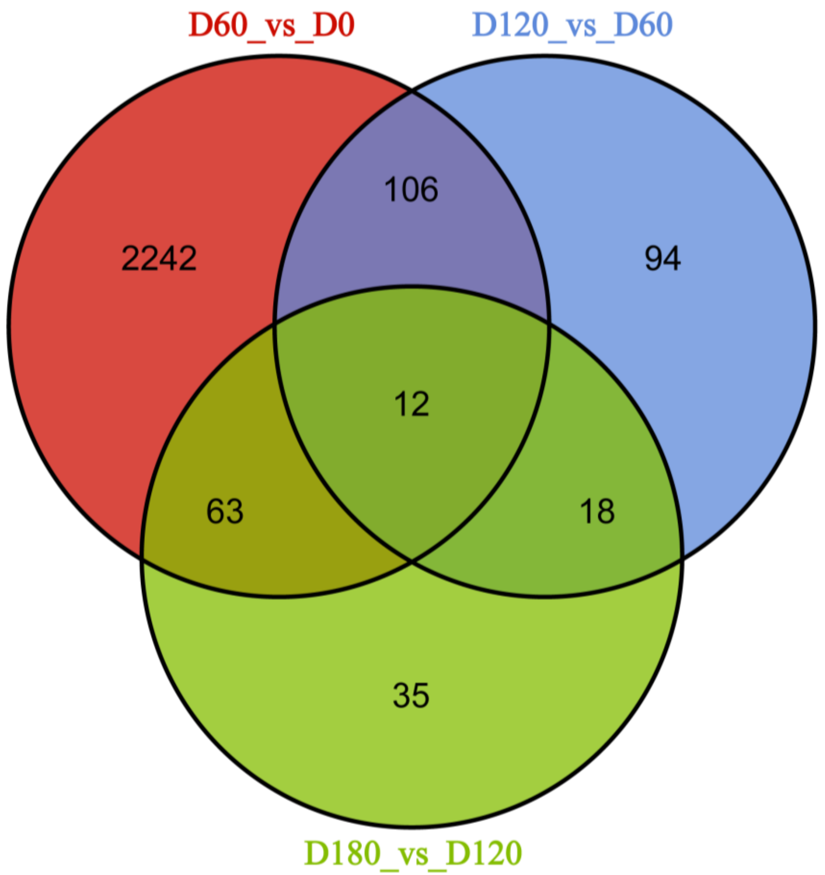

Figure S5. Venn diagram of three groups D60vsD0, D120vsD60, and D180vsD120 of landrace pig skeletal muscle.

S6

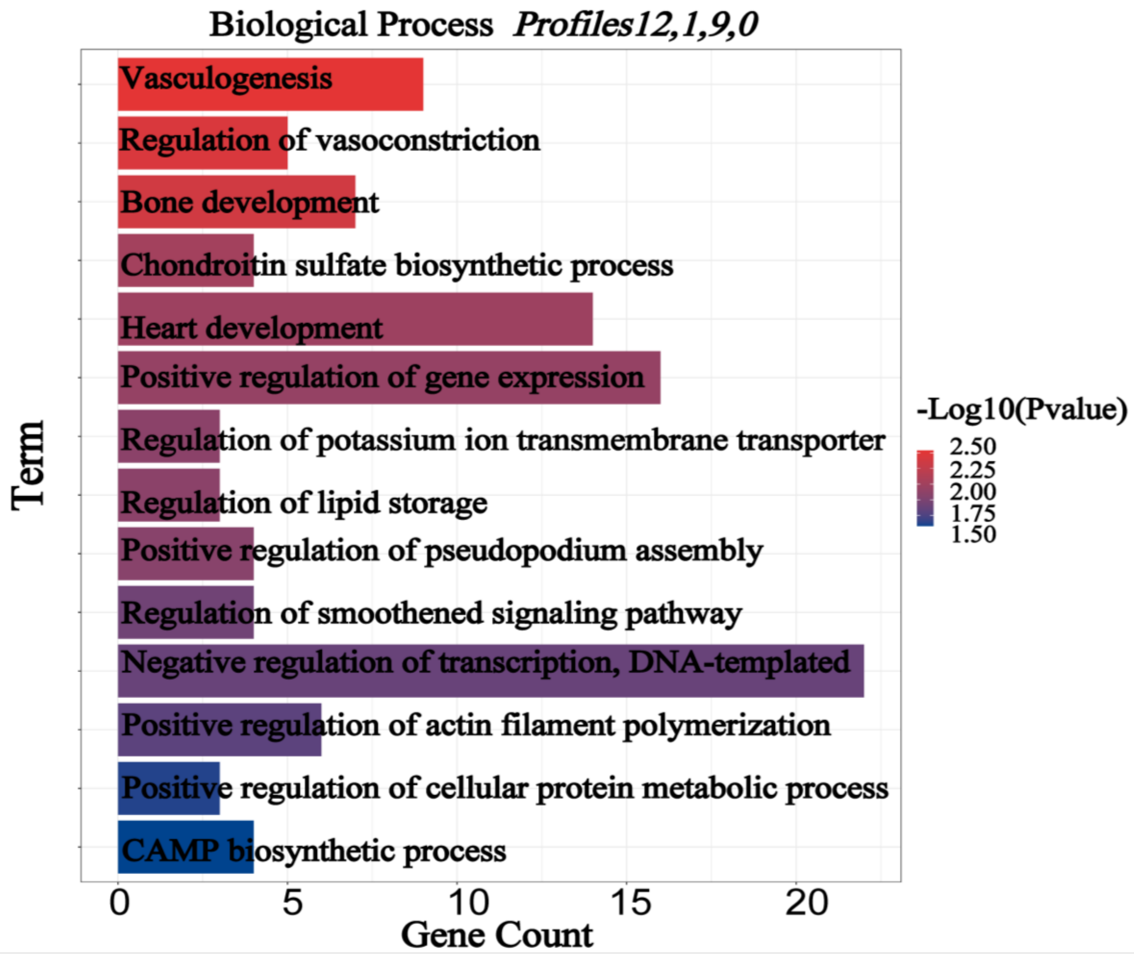

Figure S6. The biological process map of GO enrichment analysis after all genes in Profiles 12, 1, 9, and 0 was obtained after trend analysis during skeletal muscle development in Landrace pigs.

S7

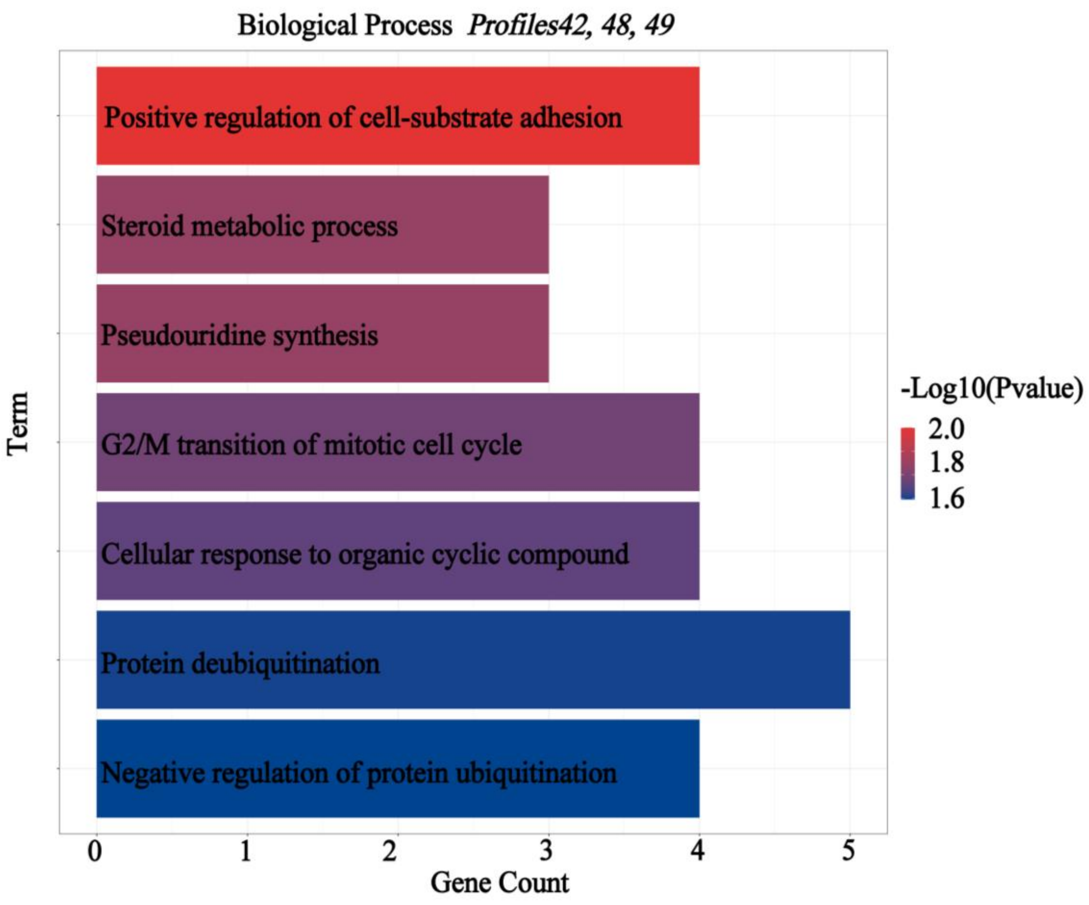

Figure S7. The biological process map of GO enrichment analysis after all genes in Profiles 42, 48, 49 and 0 was obtained after trend analysis during skeletal muscle development in Landrace pigs.

S8

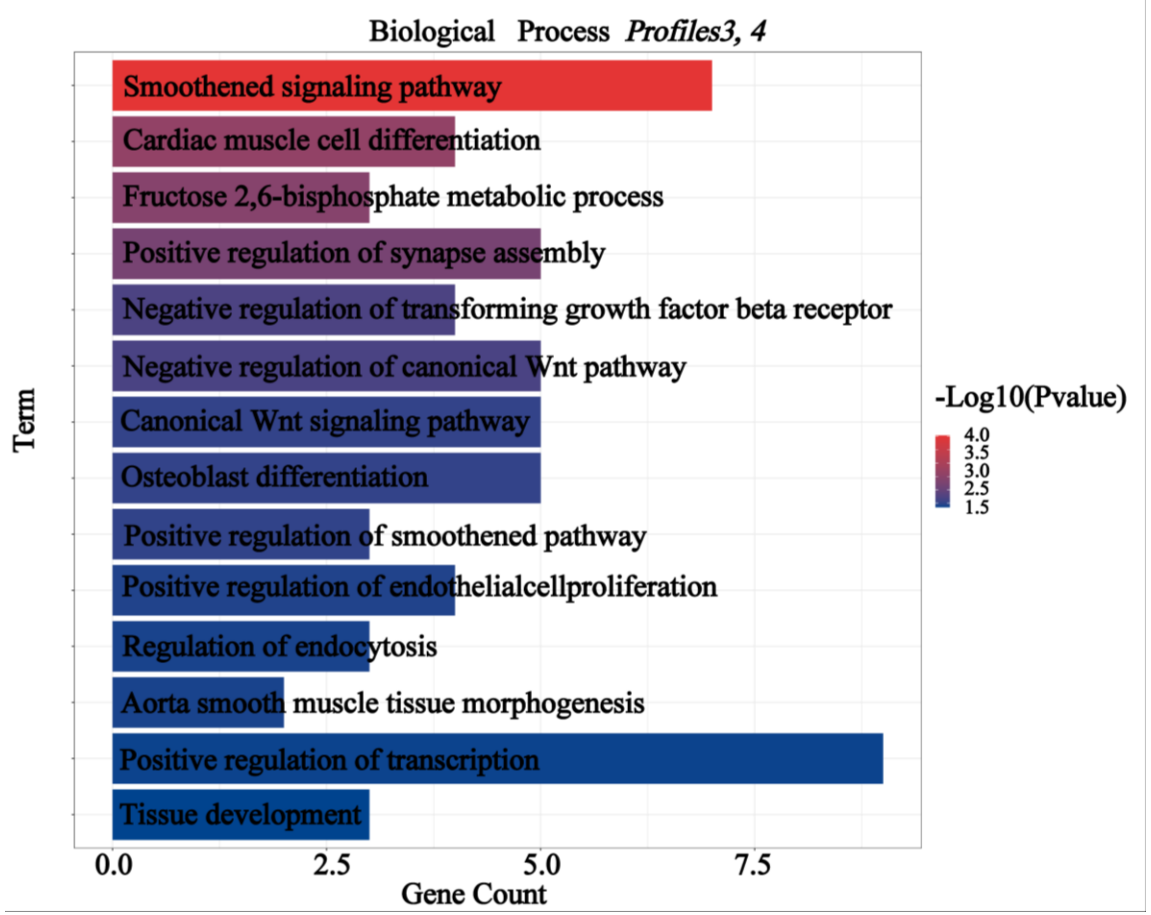

Figure S8. The biological process map of GO enrichment analysis after all genes in Profiles 3 and 4 was obtained after trend analysis during skeletal muscle development in Landrace pigs.

S9

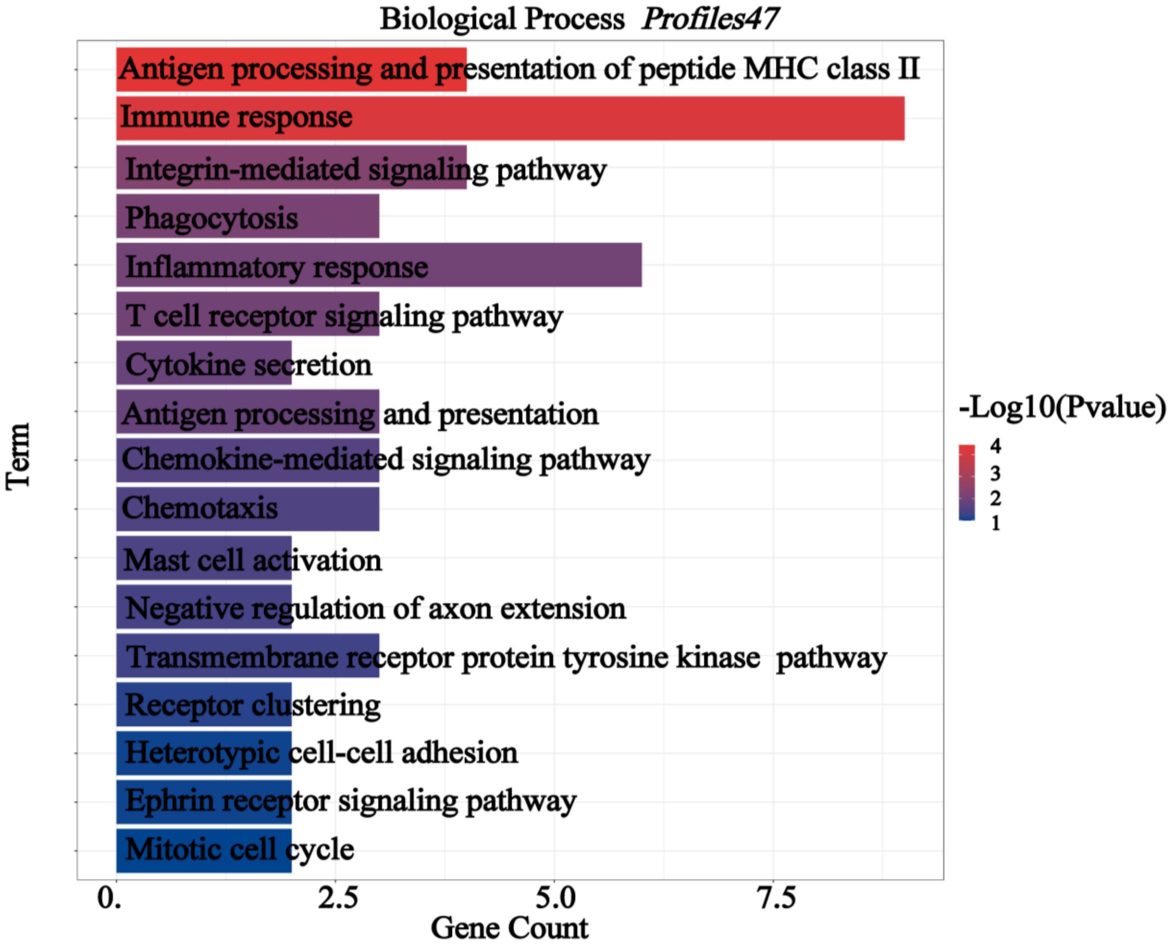

Figure S9. The biological process map of GO enrichment analysis after all genes in Profiles 47 was obtained after trend analysis during skeletal muscle development in Landrace pigs.

S10

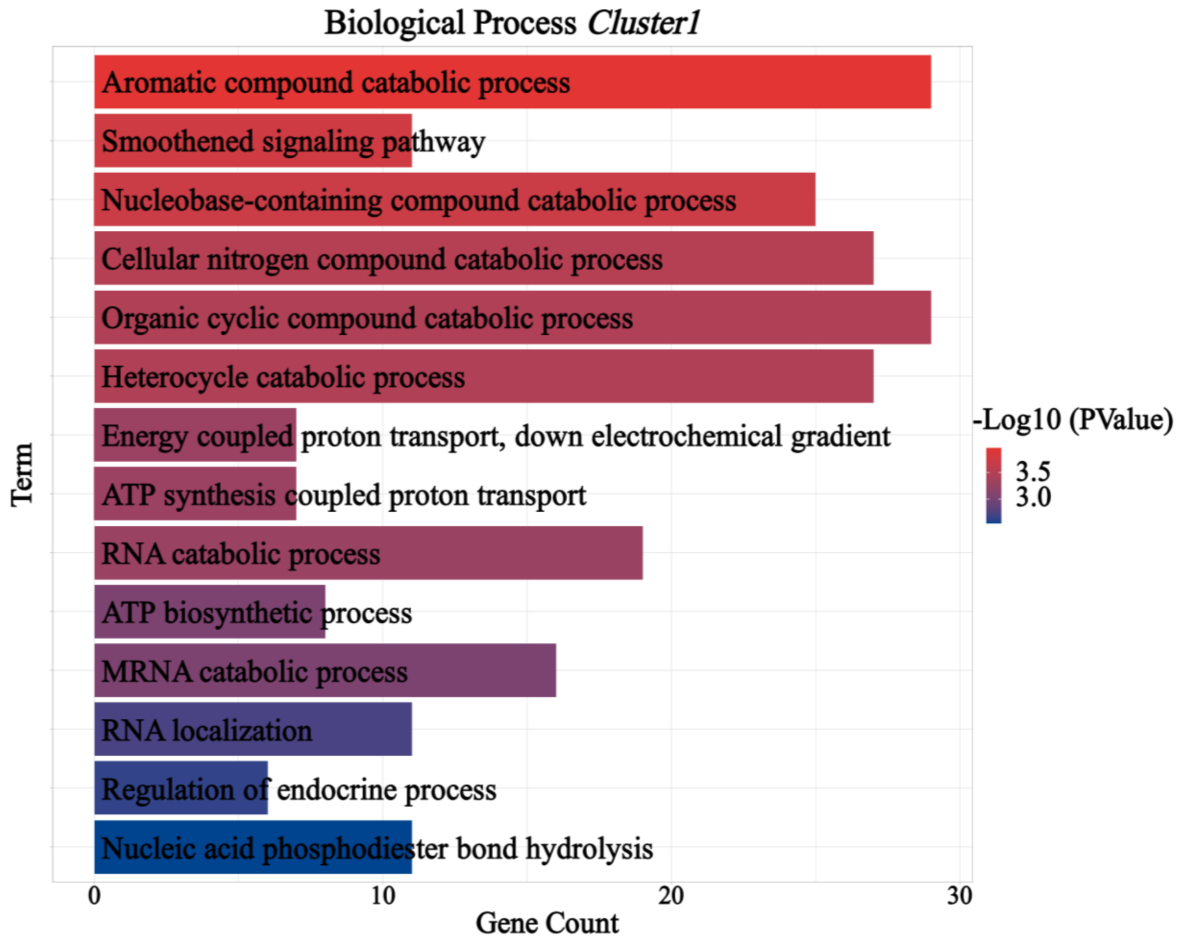

Figure S10. The biological process map of GO enrichment analysis after all genes in Cluster 1 was obtained after trend analysis during skeletal muscle development in Landrace pigs.

S11

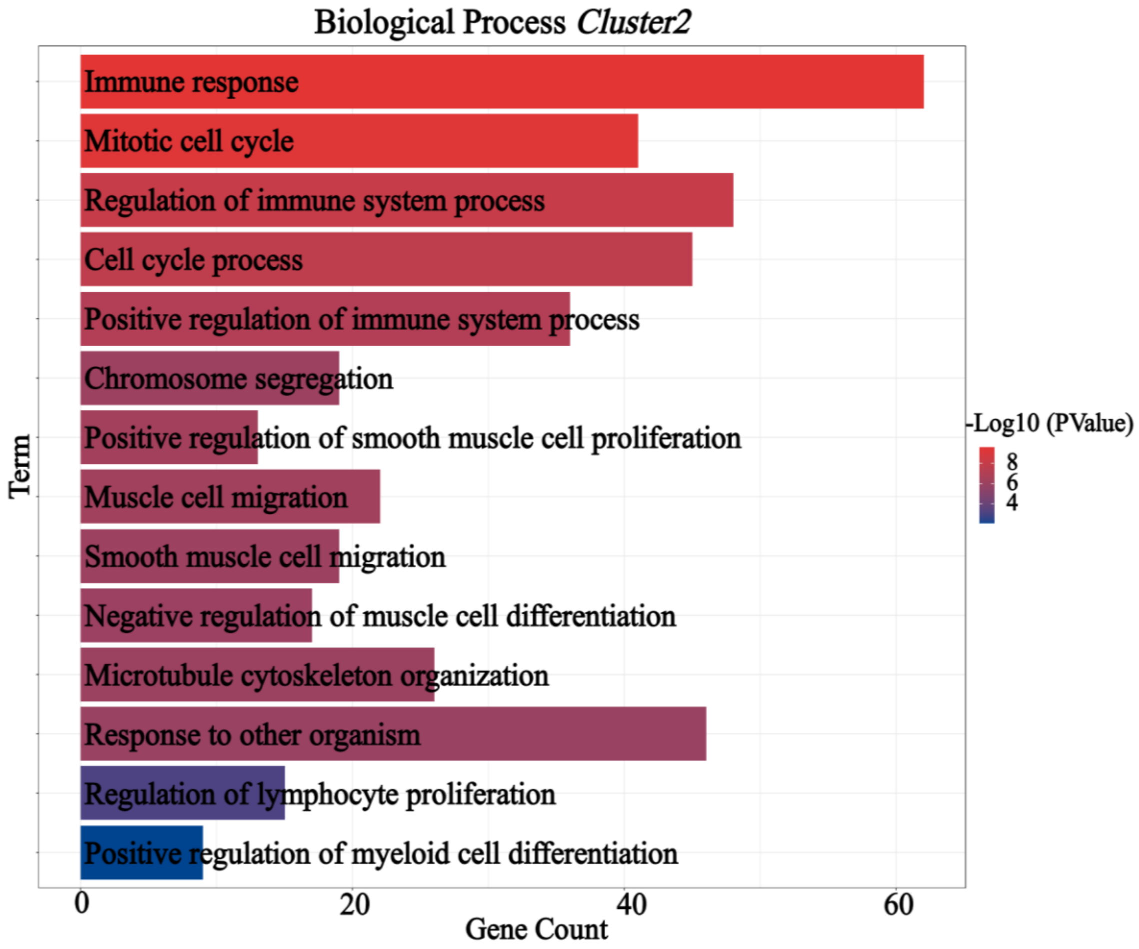

Figure S11. The biological process map of GO enrichment analysis after all genes in Cluster 2 was obtained after trend analysis during skeletal muscle development in Landrace pigs.

S12

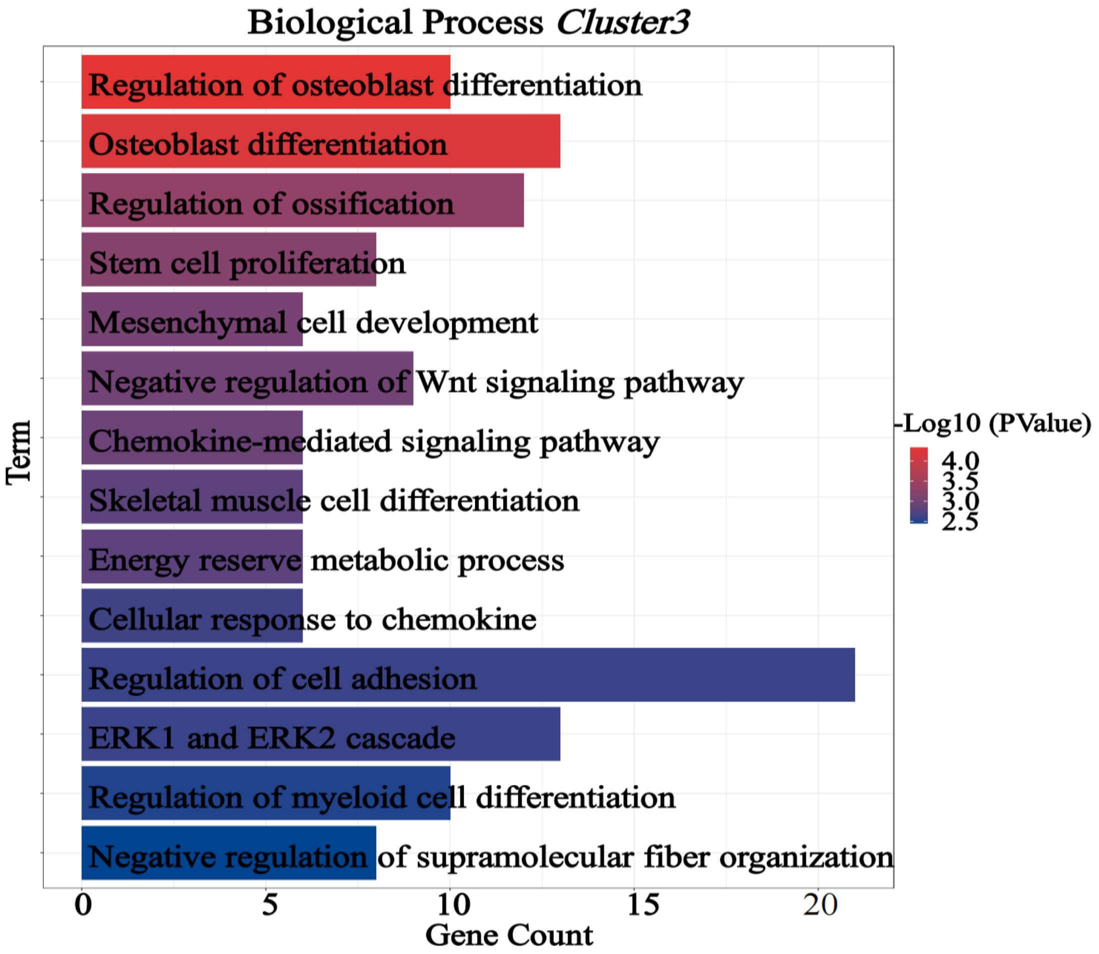

Figure S12. The biological process map of GO enrichment analysis after all genes in Cluster 3 was obtained after trend analysis during skeletal muscle development in Landrace pigs.

S13

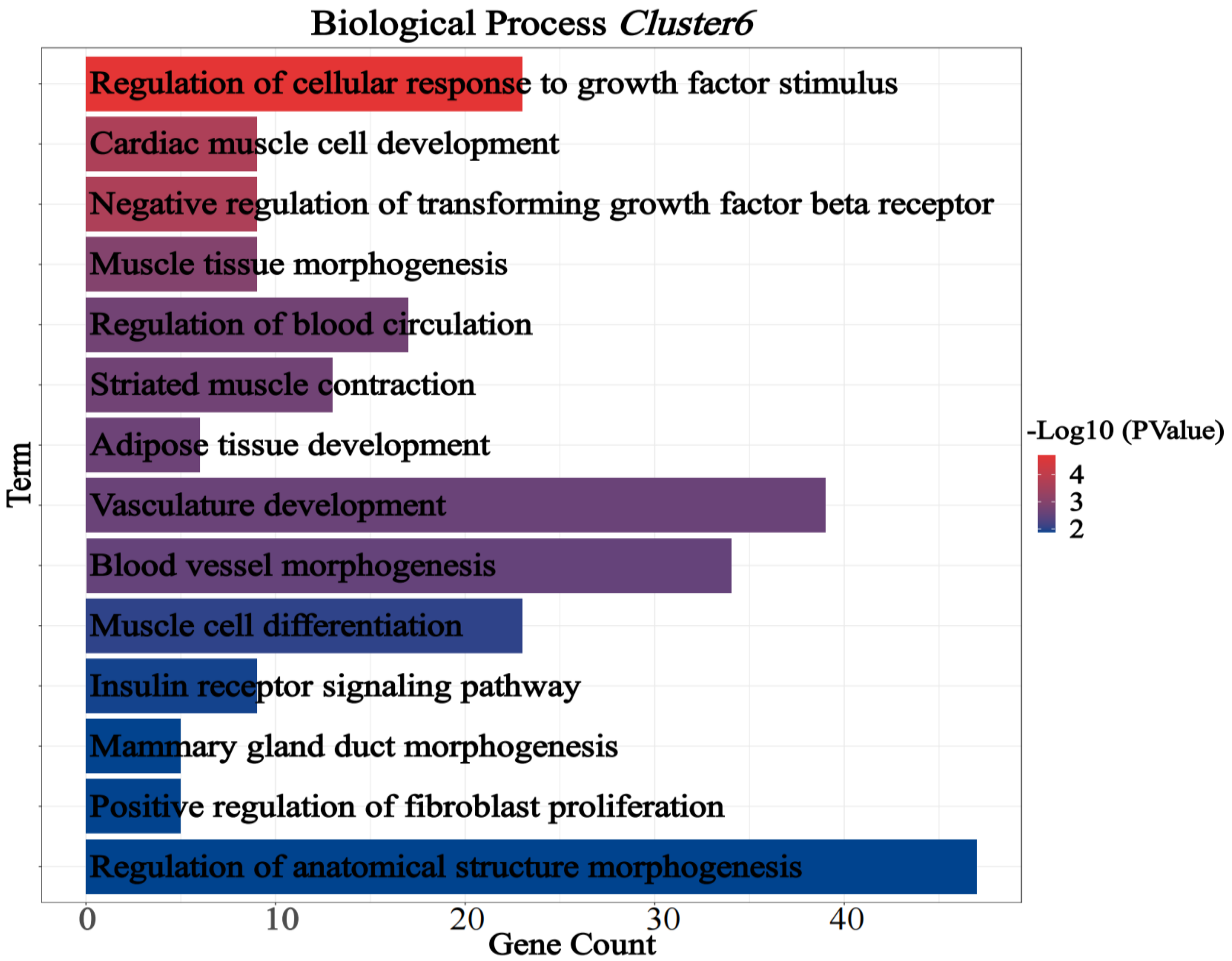

Figure S13. The biological process map of GO enrichment analysis after all genes in Cluster 6 was obtained after trend analysis during skeletal muscle development in Landrace pigs.

S14

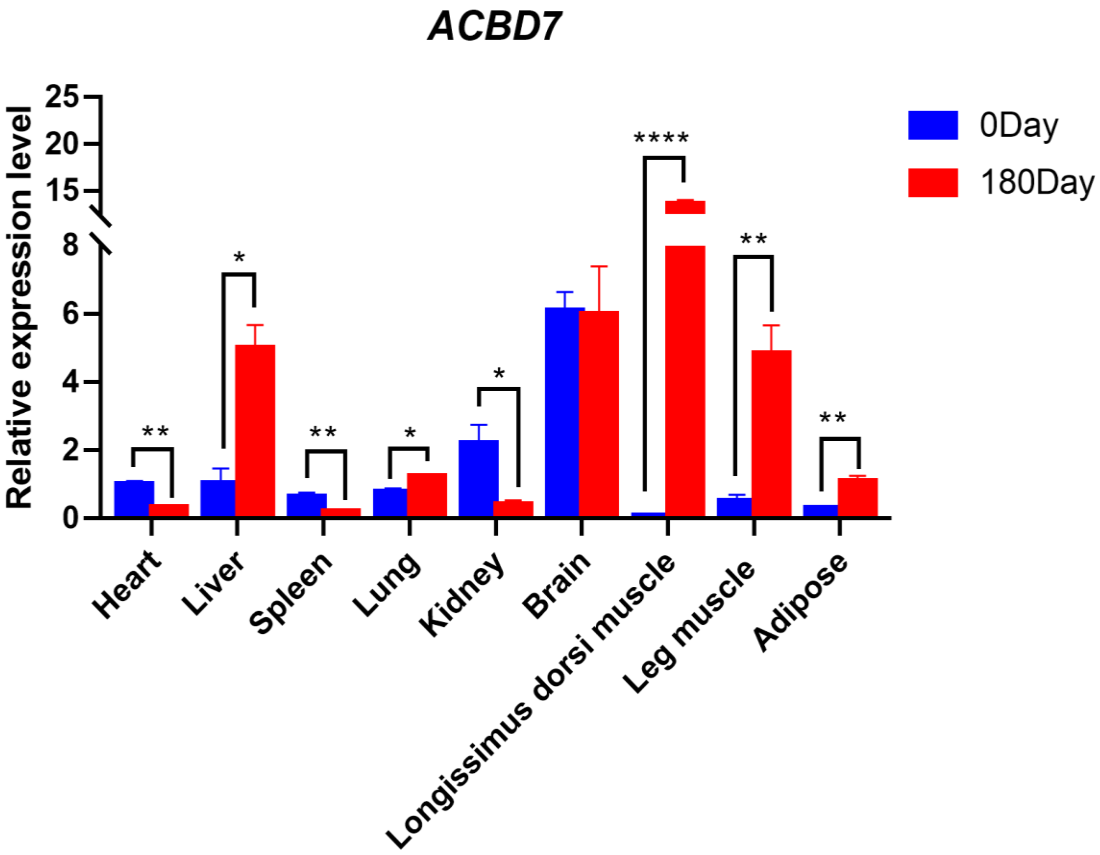

Figure S14. Expression of ACBD7 Gene in Different Tissues of Landrace pig. Note: *means significant difference ($P < 0.05$); **means very significant difference ($P < 0.01$) ; ****means very significant difference ($P < 0.0001$) .

Table S1. The summary of data generated by RNA sequencing.

Sample	Raw Reads Number	Raw Bases Number	Raw Q30 Bases Rate(%)	Clean Reads Number	Clean Bases Number	Clean Reads Rate(%)	Clean Q30 Bases Rate(%)
D0-1	44,924,186	6,738,627,900	93.06	44,010,612	6,601,591,800	97.97	93.37
D0-2	45,375,928	6,806,389,200	93.11	44,203,674	6,630,551,100	97.42	93.55
D0-3	43,455,204	6,518,280,600	93.46	42,280,234	6,342,035,100	97.30	93.73
D60-1	46,716,874	7,007,531,100	94.70	44,759,642	6,713,946,300	95.81	95.00
D60-2	46,546,304	6,981,945,600	94.79	45,345,610	6,801,841,500	97.42	95.10
D60-3	45,164,572	6,774,685,800	94.63	44,024,034	6,603,605,100	97.47	94.98
D120-1	48,016,028	7,202,404,200	94.62	46,466,284	6,969,942,600	96.77	95.01
D120-2	46,678,706	7,001,805,900	94.88	45,475,138	6,821,270,700	97.42	95.21
D120-3	46,851,850	7,027,777,500	94.79	44,986,684	6,748,002,600	96.02	95.12
D180-1	48,145,458	7,221,818,700	94.90	46,185,784	6,927,867,600	95.93	95.20
D180-2	45,734,972	6,860,245,800	94.89	44,635,138	6,695,270,700	97.59	95.19
D180-3	46,137,732	6,920,659,800	94.98	44,754,252	6,713,137,800	97.00	95.30

Table S2. ATAC-seq data statistics.

Sample ID	Raw reads	Raw Bases (G)	Raw Q30 (%)	Raw GC (%)	Clean Reads	Clean Bases (G)	Clean Q30 (%)	Clean GC (%)	Clean reads rate (%)
D0-1	192,824,860	28.92	93.74	49.84	186,839,412	22.29	97.27	50.03	96.9
D0-2	202,187,430	30.33	92.97	48.98	196,243,118	21.88	97.30	48.93	97.06
D0-3	228,890,516	34.33	92.81	49.68	222,576,452	23.51	97.54	50.01	97.24
D60-1	241,683,452	36.25	91.06	45.28	224,130,406	29.57	95.45	44.68	92.74
D60-2	118,611,082	17.79	87.49	47.68	111,748,678	10.04	95.11	47.50	94.21
D60-3	127,506,464	19.13	86.72	49.03	118,732,552	10.69	94.90	48.64	93.12
D120-1	160,902,558	24.14	88.33	47.09	149,387,592	15.80	94.77	46.34	92.84
D120-2	179,843,452	26.98	88.48	48.11	167,492,802	18.28	94.44	47.73	93.13
D120-3	158,216,190	23.73	88.4	47.1	148,204,958	15.79	94.40	46.62	93.67
D180-1	190,522,382	28.58	90.91	44.84	181,234,408	22.75	95.32	43.98	95.12
D180-2	117,259,428	17.59	85.67	46.63	112,596,366	9.52	94.91	45.97	96.02
D180-3	168,450,862	25.27	91.98	46.47	161,580,512	18.00	97.12	45.23	95.92

Table S3. Intergrative RNA-seq and ATAC-seq analysis of GO and KEGG enrichment results for overlapping genes in D60vsD0, D120vsD0, D180vsDD0, D120vsD60, D180vsD60 and D180vsD120 groups.

Group	Category	Term	PValue	Genes
D60vsD0	Biological Process	GO:0071447~cellular response to hydroperoxide	0.0002	*DAPK1, PRKD1*
D60vsD0	Biological Process	GO:0031647~regulation of protein stability	0.0008	*TRIM24, NEDD4L, PRKD1*
D60vsD0	Biological Process	GO:0043536~positive regulation of blood vessel endothelial cell migration	0.0032	*ADAM17, PRKD1*
D60vsD0	Biological Process	GO:0009887~animal organ morphogenesis	0.0221	*ATRNL1, NTN4, NTN1*
D60vsD0	Biological Process	GO:0007219~Notch signaling pathway	0.0248	*ADAM17,MAML3, ZNF423*
D60vsD0	Molecular Function	GO:0004672~protein kinase activity	0.0260	*PTK7,TRIM24, PRKD1*
D60vsD0	Molecular Function	GO:0004674~protein serine/threonine kinase activity	0.0430	*MAPK10,DAPK1, PRKD1*
D60vsD0	Cellular Component	GO:0005911~cell-cell junction	0.0042	*ADAM17,PTK7, PRKD1*
D60vsD0	Cellular Component	GO:0015629~actin cytoskeleton	0.0098	*ARHGAP32, ADAM17, DAPK1, CTNNA1*
D60vsD0	Cellular Component	GO:0005938~cell cortex	0.0216	*ARHGAP32, PRKD1*
D60vsD0	KEGG	ssc04910:Insulin signaling pathway	0.0330	*MAPK10, PDE3B, PRKAG2*
D60vsD0	KEGG	ssc04022:cGMP-PKG signaling pathway	0.0080	*PLCB4, PRKCE, PDE3B, CREB3L2, ITPR1, MYLK,*
D60vsD0	KEGG	ssc04310:Wnt signaling pathway	0.0080	*MAPK10, APC2, TCF7L2, PLCB4*
D120vsD60	Biological Process	GO:0032868~response to insulin	0.0190	*EGR1, ZBTB7B*
D120vsD60	Biological Process	GO:0035914~skeletal muscle cell differentiation	0.0220	*EGR1, EGR2*
D120vsD60	Molecular Function	GO:0001228~transcriptional activator activity, RNA polymerase II transcription regulatory region sequence-specific binding	0.0220	*EGR1, EGR2, ZBTB7B*
D120vsD60	Cellular Component	GO:0005654~nucleoplasm	0.0070	*EGR1, EGR2, CDK1, ZBTB7B, ASF1B*

Group	Category	Term	PValue	Genes
D180vsD0	Biological Process	GO:0007264~small GTPase mediated signal transduction	4.53E-04	*TIAM2, ARHGAP32, SH2D3A, RASGEF1B,*
		GO:0045766~positive regulation of angiogenesis	5.75E-04	*AKT3, ITGB8, PRKCA, PRKD1,*
		GO:0043536~positive regulation of blood vessel endothelial cell migration	0.0029	*ADAM17, PRKCA, PRKD1*
	Molecular Function	GO:0005085~guanyl-nucleotide exchange factor activity	9.85E-04	*TIAM2, SH2D3A, IQSEC1, RASGEF1B,*
		GO:0003779~actin binding	0.0040	*FMNL2, ALKBH4, CAPZB, MAP1B,*
	Cellular Component	GO:0031012~extracellular matrix	4.69E-04	*COL28A1, ADAMTSL1, SPON1, MMP14,*
		GO:0014069~postsynaptic density	0.0069	*ARHGAP32, DLG1, CAPZB, CTNND2,*
	KEGG	ssc04010:MAPK signaling pathway	0.006	*AKT3, RAPGEF2, FLNB, PRKCA, MAPT, DUSP16, MAP3K4*
		ssc04015:Rap1 signaling pathway	0.0166	*ADCY9, AKT3, PRKCA,PRKD1, ACTG1*
D180vsD120	Biological Process	GO:0060716~labyrinthine layer blood vessel development	0.0056	*HES1, CCN1*
		GO:0030513~positive regulation of BMP signaling pathway	0.0111	*HES1, CCN1*
		GO:0035914~skeletal muscle cell differentiation	0.0121	*EGR1, FOS*
	Molecular Function	GO:0005654~nucleoplasm	0.099	*EGR1, HES1, FOS*
	Cellular Component	GO:0061629~RNA polymerase II sequence-specific DNA binding transcription factor binding	0.0303	*HES1, FOS*
	KEGG	ssc04933:AGE-RAGE signaling pathway in diabetic complications	0.0448	*EGR1, DIAPH1*
		ssc04928:Parathyroid hormone synthesis, secretion and action	0.0460	*EGR1, FOS*

Group	Category	Term	PValue	Genes
D120vsD0	Biological Process	GO:0071900~regulation of protein serine/threonine kinase activity	0.0017	*UVRAG, PRKAG2, PRKAG3*
		GO:0071447~cellular response to hydroperoxide	0.0058	*DAPK1, PRKD1*
		GO:0043536~positive regulation of blood vessel endothelial cell migration	0.0097	*ADAM17, PRKCA, PRKD1*
		GO:0055013~cardiac muscle cell development	0.0202	*MYO18B, MTOR, PLEC*
	Molecular Function	GO:0005524~ATP binding	1.36E-05	*FBH1, PRKAG2,ATP1A1, MYLK3,NMNAT3, MAPK10, MYO3B, PRKD1, CDK13,*
	Cellular Component	GO:0015629~actin cytoskeleton	0.0488	*ADAM17, MYO3B, DAPK1, CTNNA1,*
	KEGG	ssc04919:Thyroid hormone signaling pathway	0.0020	*STAT1, GATA4, PRKCA, ATP1A1, PFKM, RXRG, MED12L, MTOR*
		ssc04152:AMPK signaling pathway	0.0021	*CPT1A, SCD5, PRKAG2, PFKM, MTOR, ACACA, FBP2, PRKAG3*
		ssc04935:Growth hormone synthesis, secretion and action	0.0303	*MAPK10, STAT1, PRKCA, ADCY8, MTOR*
D180vsD60				*DGKB, KPNA5,DGKB, KPNA5,RORB,GALNT1, KCTD8,*

Table S4. The fluorescence intensity of PK15 cells transfected for 24 h, 48 h, and 72 h was analyzed using Image J software.

Times	Mean fluorescence intensity (Mean)
24h	35.79±9.57[b]
48h	78.50±12.48[a]
72h	88.05±11.94[a]

Note: a,b indicates data in the same row with completely different lowercase letters indicates significant difference (p < 0.05), and data with any same lowercase letters or no letters indicates no significant difference (p > 0.05).

Table S5. The primer sequences for RT-PCR

Genes	Primer sequences(5’→3’)		Product /bp	GenBank accession No.
TMEM220	F:ACCCTCTTGTCACAGGTAGC	R:TGACACAGGCTCATCCATGC	187	XM_003131997.5
THBS1	F:GGATGGTCGAGGTGATGCTT	R:CTGATGGCGTACAACCCAGT	178	NM_001244536.1
RHOB	F:GAGAACATCCCCGAGAAGTGG	R:ACTCGAGGTAGTCGTAGGCTT	199	NM_001123189.1
PLK3	F:TCTGTGCCCTGAGAAACTGC	R:TTGAAAAGCACAGCCACACG	166	XM_003128058.4
MYL9	F:CCTCAGGCTTCATCCACGAG	R:ACCGTGTTTGAGGATGCGAG	158	NM_001244472.1
LRTM1	F:TTTCCATTCCCTCCCACAGC	R:GGCATGGATTCTAGGAGGGC	159	XM_021068978.1
IL18	F:GCTGCTGAACCGGAAGACAA	R:AAACACGGCTTGATGTCCCT	192	NM_213997.1
HES1	F:CAACGCCATGACCTACCCTG	R:CGAATACCTTTGCCGCCTCT	200	NM_001195231.1
FOS	F:TCAGAGCATTGGCAGAAGGG	R:GTGTGTCAGTCAGCTCCCTC	134	NM_001123113.1
CCN1	F:CACCAATGACAACCCCGACT	R:ACTTGGGCCGGTACTTCTTC	182	NC_010446.5
BMP2	F:TTCTTAGACGGTCTGCGGTC	R:GCAACTCAAACTCGCTGAGG	180	NM_001195399.1
ACBD7	F:TGAAGACCAGGCCAGATGAC	R:GTTCCAAGCTTCCCACTTAGC	134	XM_003357745.4
MyHC-I	F:GGCCCCTTCCAGCTTGA	R:TGGCTGCGCCTTGGTTT	63	L10129.1
MyHC-IIa	F:TTAAAAAGCTCCAAGAACTGTTTCA	R:CCATTTCCTGGTCGGAACTC	100	U11772
MyHC-IIb	F:CACTTTAAGTAGTTGTCTGCCTTGAG	R:GGCAGCAGGGCACTAGATGT	80	U90720
MyHC-IIx	F:AGCTTCAAGTTCTGCCCCACT	R:GGCTGCGGGTTATTGATGG	76	U90719.1
CD36	F:GGGTTAAAACAGGCACGGAAG	R:GCACCATTGGGCTGTAGGAA	199	NM_001044622.1
FABP4	F:CATTAGATCAGAAAGTACCT	R:ATCTAAGGTTATGGTGCTCT	112	NM_001002817.1
ATGL	F:CATCCGTGGCTGCCTGGTGAA	R:CCTGGCGGCGAAGTGGGTTAT	121	EU373817.1
β-actin	F:ACGTCGCACTGGATTTCGAG	R:TGTCAGCAATGCCAGGGTAC	282	NM_205518.2
